# Space-time confounding adjusted determinants of child HIV/TB mortality for large zero-inflated data in rural South Africa

**DOI:** 10.1016/j.sste.2011.07.001

**Published:** 2011-12

**Authors:** Eustasius Musenge, Penelope Vounatsou, Kathleen Kahn

**Affiliations:** a MRC/Wits Rural Public Health & Health Transitions Research Unit, School of Public Health, Faculty of Health Sciences, University of the Witwatersrand, Johannesburg 7 York Road, Parktown 2193, Johannesburg, South Africa; b Biostatistics and Epidemiology Division, School of Public Health, Faculty of Health Sciences, University of the Witwatersrand, Johannesburg 7 York Road, Parktown 2193, Johannesburg, South Africa; c Swiss Tropical and Public Health Institute, Socinstrasse 57, 4051 Basel, Switzerland; d Centre for Global Health Research, Umeå University, Umeå, Sweden; e INDEPTH Network, Accra, Ghana

**Keywords:** Spatiotemporal confounding, HIV/TB, Zero inflated, Over-dispersed, Agincourt rural South Africa

## Abstract

South Africa is experiencing a major burden of HIV/TB. We used longitudinal data from the Agincourt sub-district in rural northeast South Africa over the years 2000 to 2005. A total of 187 HIV/TB deaths were observed among 16,844 children aged 1–5 years coming from 8,863 households. In this paper we used Bayesian models to assess risk factors for child HIV/TB mortality taking into account the presence of spatial correlation. Bayesian zero inflated spatiotemporal models were able to detect hidden patterns within the data. Our main finding was that maternal orphans experienced a threefold greater risk of HIV/TB death compared to those with living mothers (AHR = 2.93, 95% CI[1.29;6.93]). Risk factor analyses which adjust for person, place and time provide evidence for policy makers that includes a spatial distribution of risk. Child survival is dependent on the mother’s survival; hence programs that promote maternal survival are critical.

## Highlights

► Problem of Bayesian MCMC inversion of very large data in estimating the spatial random effects known as the “Big N”. ► Use of flexible exponential family link functions to exponential family predictors to avoid identifiability related problems. ► South Africa’s HIV/TB burden in children and efficient modeling thereof. ► Addresses space-time confounding in multivariate analyses of very large data structures. ► Estimates useful for policy formulation and modeling which results in risk maps which are user friendly.

## 1 Introduction

The years 2000–2005 mark the middle of the United Nation’s Millennium Development Goals (MDG) set out by the 192 member states and 23 international organizations (Sachs and McArthur, 2005). Goal 4.1 aims at reducing child (under five) mortality by two-thirds over the years 1990 and 2015. The sub-Saharan African region has the greatest burden of disease and highest mortality of HIV/TB. In 1990 the sub-Saharan Africa region had 184 child deaths per every 1000 live births, by the year 2006 27 of the countries in the region had made no progress in reducing child deaths (UNDG, 2008). Globally, 2.1 million children were living with HIV/TB in the year 2008 of which 90% were from the sub-Saharan region (UNAIDS, 2009). South Africa’s HIV/TB orphans in 2008 were about 1.4 million, the highest absolute number globally (UNAIDS, 2008). In 1998, the year Demographic and Health Surveys were started in South Africa, there was an under five mortality rate (U5MR) of 59.4 deaths per 1000 live births, and aims to reduce this to 20 per 1000 by 2015 (UNESCO, 2007). The main cause of child deaths is HIV epidemic, contracted mainly through mother-to-child-transmissions (MTCT) with over 70,000 new infections yearly (UNICEF, 2009).

Public health priorities such as disease control intervention are made difficult in many developing countries due to lack of good data mainly due to poor resources, poor management and inadequate skills. The analyses of public health data often build from the statistical notion of each person having a risk or probability of contracting a disease. The main goals in analyses are to identify and quantify any exposures, behaviours and characteristics that may modify an individuals’ or a population’s risk. However HIV/TB interventions are hampered by a complex interlink of social, behavioural, biological and spatial determinants.

Many articles have been published on HIV/TB mortality in Sub-Saharan Africa. The issues focused mainly on the impact of epidemics, assessing risk factors, measuring impact of interventions and identifying high risk clusters. A study done in rural Senegal showed a decreasing trend in child mortality over a 37 year period (Delaunay et al., 2001). Spatial analyses on 39 rural villages in Burkina Faso under the Health and socio-Demographic Surveillance System (HDSS) over a 9 year period, showed that village and distance from the health facility where among the risk factors for child mortality (Kynast-Wolf et al., 2002). A seasonal mortality trend was also disclosed by the same authors in another study of the same HDSS (Becher et al., 2004). A Tanzanian study used spatial modeling techniques and found that there is a need to adopt both a group-based and a place-based approach in identification of high-risk HIV groups for intervention (Msisha et al., 2008). In South Africa rural Kwazulu-Natal a study found that prevalence of HIV was associated with ethnicity, urban status and unemployment based on over 11,000 persons in 700 enumeration areas (Kleinschmidt et al., 2007). In north east South Africa the relative risk (RR) of HIV and TB has increased 10-fold over an 11 year period; reaching 32.3 from 3.2 for the period 2001–2003 compared to 1992–1994, (Kahn et al., 2007). Covariates that have mainly been used to model child mortality are: HIV exposure, poor maternal health, inadequate infant care, increased exposure to infections, increased mortality, immune system abnormalities, poor nutrition, reduced breastfeeding and antiretroviral exposure (Filteau, 2009). Methods applied in the analyses were mostly non-Bayesian based, which are limited in catering for large datasets, time and spatial random effects (Pacheco et al., 2008).

In epidemiological research, two study designs have been used for follow-up studies: time series studies, that estimate acute risk associated with short-term exposure and cohort studies, that estimate chronic effects associated with long term exposure. These approaches have been criticized for potential confounding by time varying covariates and individual risk factors or household level traits, respectively (Greven et al., 2009). Failure to account for extra-variation induced by temporal and spatial autocorrelation can lead to an understatement of the uncertainty of the risk of mortality (Burnett et al., 2001). Several approaches can be used to address confounding, at the design level – randomization, during the study – use of experts and validated instruments and during analyses – multivariable regression, standardization and stratification (Greenland, 1989). Bayesian and spatial statistical techniques can be used to evaluate differences in rates observed from different geographical locations, separate noise from patterns and identify disease clusters.

However, these methods have been rarely used in HIV/TB research to assess the diseases’ impact on populations and their areas of residence. In this paper, we used Bayesian models to assess risk factors of child HIV/TB mortality taking into account the presence of spatial correlation. The modelling procedure adjusts for the household level spatial random effects for child HIV/TB mortality. Some related earlier work has looked at all-cause mortality adjusting for village (areal) level random effects (Sartorius et al., 2011, 2010). Our findings can aid in understanding geographically adjusted risk factors of child HIV/TB mortality in a rural South African cohort with sparse discrete outcomes.

## 2 Methods

### 2.1 Agincourt Health and Socio-demographic Surveillance System (HDSS) data

**Fig. 1:**
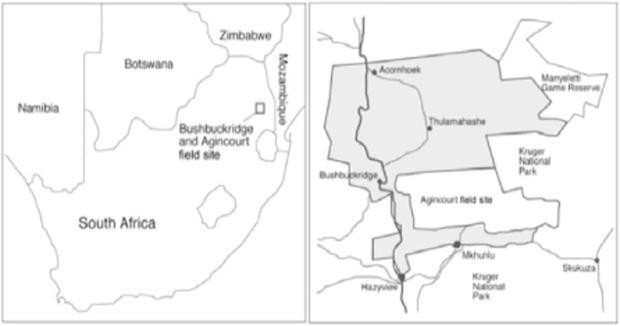
Agincourt HDSS location North-East of South Africa. Source Kahn et al., 2000.

The Agincourt HDSS was set up in 1992 in a rural sub-district in Bushbuckridge Municipality of Mpumalanga Province, northeast South Africa. The location was selected mainly due to availability of several clinics and its lack of infrastracture (Tollman, 1999). The Agincourt subdistrict had a population of over 70,000 persons, living in approximately 12,000 households scattered in 21 villages. The area covered 432 square kilometres, with 34.2 km as the maximum distance between two households (Fig. 1). Information at individual level (infants, children and adults) is collected once every year including vital events updates, that compile migration, health, demographic, mortality and fertility data (Kahn et al., 2007).

Special modules are run periodically such as socio-economic status and food security. Cause of death data are obtained through verbal autopsies conducted on every recorded death by trained lay fieldworkers (Kahn et al., 2000). The cause of death is independently determined by two or three medical doctors and classified according to the World Health Organization’s International Classification of Diseases (ICD10). Every household is geo-coded thus enabling spatial analysis at household as well as at village level. Agincourt HDSS longitudinal data are correlated in time and space (spatiotemporal), since they are collected yearly on the same population. This makes it difficult to analyze the data due to their collinearity and also their large size.

### 2.2 Outcome and explanatory variables

The child mortality independent variables were selected in accordance with the approach proposed by Rutstein (2000) who mentions six main risk factors for child mortality: socio-economic status, nutritional status, use of health facilities, environmental health conditions, maternal factors and infant feeding (Rutstein, 2000). The persons included in the study were all the children aged between 1 and under 5 years who lived or had lived in the Agincourt HDSS between January 2000 and December 2005. The explanatory variables used were: child’s gender, child’s and mother’s nationality, mother’s age, parents’ dead or alive, gender of household head, antenatal clinic visit, mother’s parity at pregnancy and birth, cumulative deaths in household, number of household dwellers, number of deaths in household and mother’s total live births. The individual child’s age was treated as an offset variable.

The outcome variable was death due to HIV and or tuberculosis (TB) determined by the WHOs ICD10 verbal autopsy codes A16–A19^1^ for TB and B20–B24^2^ for HIV. The outcome death was treated as a discrete (count) variable since this provides epidemiologically better estimates of prevalence and relative risk compared to the odds ratio which results from treating it as binary outcome (Barros and Hirakata, 2003; Fekedulegn et al., 2010). Treating the outcome as such allowed us to employ the Poisson and Negative Binomial which yields more epidemiologically plausible relative risks for these cohort data. These models which are members of the exponential family have a log link function which has less identifiability issues in the generalized linear model compared to the logit link function which results from treating the outcome as binary and using logistic regression techniques (Agarwal et al., 2002).

### 2.3 Spatiotemporal zero inflated Poisson and Negative Binomial models

Agincourt child HIV deaths data had several problems that made it difficult to use standard statistical procedures: over dispersion, caused by unobserved heterogeneity or spatio-temporal correlation and data collected over large numbers of locations with zero deaths. Conventional statistical methods applied to spatiotemporal data often underestimate the standard error and thus the statistical significance is overestimated (Cressie, 1993). Bayesian analyses of multilevel models for complex geostatistical data can be done with the aid of Markov Chain Monte Carlo (MCMC) parameter estimation procedure (Banerjee et al., 2004). Bayesian geostatistical models for temporal count data with large number of zeros have been proposed by Fernandes et al. (2009). We employ the above methodology combined with semi-parametrically structured geo-additive predictors, to identify the determinants of HIV/TB related mortality in Agincourt HDSS data.

Geo-additive spatiotemporal zero inflated Poisson (ZIP) as well as Negative Binomial (ZINB) models (see Appendix A) were used in the analysis. The models have many parameters and are hierarchical (multilevel), thus we resort to full Bayesian inference with the computationally efficient MCMC techniques. Multiple variable models were used to assess the effect of different covariates in the presence and absence of geographical heterogeneity (Gosoniu et al., 2008). We investigated the presence of over-dispersion (when δ→∞ it is absent) and zero inflation (when θ=0, it is absent) (Fahrmeir and Echavarria, 2006). Spatial dependence $f_{\mathrm{spat}}(s)$ was modelled by assuming that the random effects follow a zero mean stationary multivariate Gaussian random field (GRF) with spatial variance $\sigma_{\mathrm{spat}}^{2}$ and isotropic Matern correlation functions. For the exponential correlation which is a special case of the Matern function, the multivariate GRF $f_{\mathrm{spat}}(s)∼\mathrm{MVN}(0\text{,}\sum )$ has variance–covariance matrix ∑=σspat2exp[-ρ^dij], where $d_{\mathrm{ij}}$ is the Euclidean distance between households *i* and $j\text{,}\sigma_{\mathrm{spat}}^{2}$ is the geographic (spatial) variability known as the sill and ρ^=maxij‖si-sj‖/0.001 is the empirically estimated rate of spatial correlation decay (Brezger et al., 2005). Multivariate Gaussian random field (GRF) priors $f_{\mathrm{spat}}(s)=p(\beta^{\mathrm{spat}}|\sigma_{\mathrm{spat}}^{2})\propto |\sum {|}^{-\frac{1}{2}}\exp\left(\frac{-1}{\sigma_{\mathrm{spat}}^{2}}{\left(\beta^{\mathrm{spat}}\right)}^{\prime }\sum^{-1}\beta^{\mathrm{spat}}\right)$, where $\beta^{\mathrm{spat}}$ is the vector of household specific structured spatial coefficients, whereas the non-structured utilize the independent and identically distributed (i.i.d) Gaussian priors with Inverse Gamma (IG) hyper-priors (Brezger and Lang, 2006).

Bayesian based model fitting requires the inversion of the variance–covariance matrix (∑) with the same size as the number of geo-locations (households). Due to the large number of observations in our dataset, the estimation of model parameters becomes unstable and unfeasible; this problem is popularly known as the “big N”. We addressed this problem by using low rank kriging to approximate the stationary Gaussian random field (Kamman and Wand, 2001). Firstly the spatial correlation decay ρ^ is estimated from a “representative” subsample obtained using the mini-max space filling criteria, hence only parameter $\sigma_{\mathrm{spat}}^{2}(1/\tau_{\mathrm{spat}}^{2})$ needs to be estimated. This reduces the computational burden since the spatial decay is fixed and the large Euclidean distance matrix is also constant, resulting in estimation of the spatial variance and parameter vector of household spatial effects.

Full Bayesian inference was done via MCMC simulation based on updating full conditionals of single parameters or blocks of parameters, given the rest of the data. Gibbs sampling was used for closed conditionals and for the numerically intractable a slightly modified form of the Metropolis-Hastings (MH) algorithm based on iteratively weighted least squares (IWLS) (Casella and George, 1992; Banerjee et al., 2004). A total of 55,000 iterations on a single chain were run with the first 5000 discarded as burn-in and to reduce auto-correlation every 25th value was taken to form a posterior sample of 2000. MCMC convergence was assessed using diagnostic procedures proposed by Raftery and Lewis (1992), Geweke and Minneapolis (1991) and Heidelberger and Welch (1983). These convergence diagnostics techniques were done in the R-package, “convergence diagnosis and output analysis” (CODA) (Plummer et al., 2006). Additionally a graphical assessment was done using trace-plots, histogram density plots and auto-correlations of parameters and the summary statistics assessing the closeness of the mean and median posterior estimates (Nylander et al., 2008).

### 2.4 Goodness of fit and best model selection

The deviance information criteria (DIC) was used in determining goodness of fit of the proposed models, this can be defined as “classical estimate of fit, plus twice the effective number of parameters” (Spiegelhalter et al., 2002). This is a technique for Bayesian model selection for MCMC derived posteriors. The procedure works on the assumption that the posterior distribution is multivariate normal and the un-standardised deviance is given as: *D*(*Ω*) = −2 log(*p*(*y*∣*Ω*)), where *y* are the data, *Ω* are the unknown parameters of the model, *p*(*y*∣*Ω*) is the likelihood. After addition of the standardising constant *C* = 2 log *f*(*y*) we get the Bayesian or saturated deviance D(Ω)=-2log(p(y|Ω))+C. The expectation of this deviance $\overset{¯}{D}=E^{\Omega }[D(\overset{¯}{\Omega })]$ measures how well the model fits the data, the smaller it is the better. The measure of model complexity or parsimony is the effective number of parameters; $p_{D}=\overset{¯}{D}-D(\overset{¯}{\Omega })$, where $\overset{¯}{\Omega }$ is the expectation of Ω the smaller it is the more parsimonious. From these measures we get the deviance information criteria; $\text{DIC}=p_{D}+\overset{¯}{D}$ and upon substitution of $\overset{¯}{D}$ we get $\text{DIC}=2p_{D}+D(\overset{¯}{\Omega })$, which penalises on complexity and lack of fit. Selection of a better model based on the DIC basically favours models with the smaller values.

The data extraction was done using Structured Query Language (SQL). Preliminary data analyses, trend analyses, Kaplan-Meier survival graphs and data management were done using STATA 10.0 (StataCorp, 2007). The Bayesian MCMC analysis was done using a software package called BayesX (Belitz et al., 2009). This package utilises computationally efficient C++ algorithms (Press, 2002). The software ran on a quad core processor with 4.0 gigabytes of random access memory (RAM) within a Linux environment. The routines used to estimate the parameters of the models via MCMC which perform Gibbs sampling and Metropolis Hastings within Gibbs sampling. Multivariable analyses were performed to determine patterns for HIV/TB mortality adjusting for possible household confounders. Posterior risk maps were produced using mapping packages in R (R-cran, 2010).

### 2.5 Ethical clearance and informed consent

The Agincourt HDSS site was granted ethical clearance by the University of the Witwatersrand’s Committee for Research on Human Subjects (No. 960720). This work was also granted ethical clearance by the University of the Witwatersrand’s Committee for Research on Human Subjects (M081145). Verbal informed consent was obtained when the census rounds were conducted and also when verbal autopsy data were collected from a close relative of the deceased.

## 3 Results

### 3.1 Exploratory analysis and descriptive statistics

The data used were for children aged 1–5 years residing in Agincourt HDSS from 2000 to 2005 whose geo-location data were available. These totalled 16,844 children from 8863 households, with a range of 138–835 households across the 21 villages. A total of 187 deaths were HIV/AIDS and Tuberculosis related from 59,448.15 person years yielding a 1–5 years mortality rate (1–4MR) of 3.15 deaths per 1000 person years. There were slightly more females than males (51% vs 49%) and 37% were formerly Mozambican in origin. Mothers had an average age of 29 years, 65% were South African and about 1% had deceased compared to 2% of the males. The women in the area on average visited the antenatal facility four times during their pregnancy and 34% were heads of their households.

**Fig. 2:**
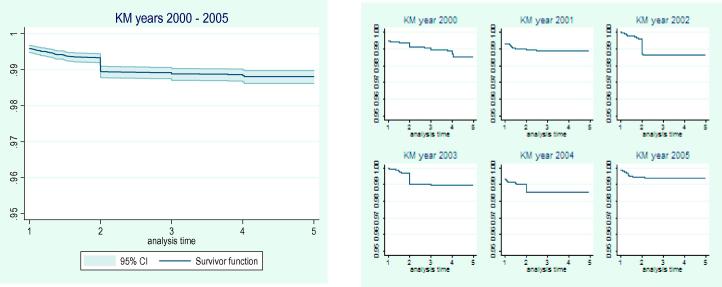
Kaplan–Meier (KM) survival function over the years 2000–2005 (left) and year specific Kaplan–Meier (KM) curves (right).

An investigation of the survival experience using the Kaplan–Meier (KM) curves (Fig. 2, left) showed differences in the survival experiences over the years 2000–2005. We assessed the differences in survival experiences over the years using the generalized Fleming–Harrington test weighted for later years failures (p = 0.048) (Fleming and Harrington, 1984). However there was no significant trend over the years (*p* = 0.728). This procedure of stratifying over the years was used to assess for the possibility of temporal random effects.

**Fig. 3:**
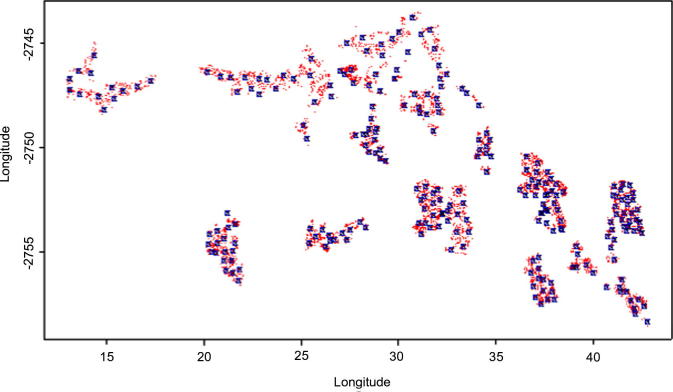
Knots fitted using the mini–max space filling criteria from Agincourt households 2000–2005.

The year specific child 1-4MRs per 1000 person years from 2000 to 2005 were 3.57, 2.41, 3.33, 2.54, 4.44 and 1.55, respectively, consistent with the KM curves (Fig. 2, right) for the respective years. There was a statistically significant difference in the 2002 mortality rates compared to 2000 from the spatial and non-spatial analyses (see Table 2). The knots used in the low rank kriging were fit using the mini-max space filling criteria, Fig. 3 shows distribution of 50 knots. The small faint (red)^3^ dots are the locations and the squares are the selected 50 knots, since the ρ^ (rate of spatial correlation decay) is fixed the number of knots only impacts on the computational speed and not on the accuracy of the estimates. The ZIP model was used to cater for zero inflation and to cater for both the excess zeros and over-dispersion, the ZINB model was used. The zero inflated Negative Binomial spatial temporal model showed an over-dispersion estimate *δ* = 1.79 ± 1.87[95% CI(0.041; 6.935)] and the zero inflation estimate was *θ* = 0.077 ± 0.062[95% CI(0.010; 0.217)]. These values show that there is some over-dispersion and zero inflation, thus ignoring these entirely in the modelling may cause model instability.

### 3.2 Spatiotemporal confounding adjusted determinants of child HIV/TB mortality

**Fig. 4:**
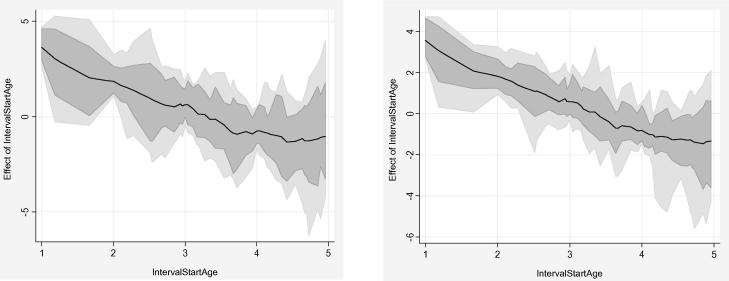
Non-spatial ZINB with AR(1) baseline hazard (left) and multivariable spatial ZINB with AR(1) baseline hazard (right).

Multivariable analysis was done using non-spatial and spatial models for child specific variables, maternal variables and household variables. Bayesian semi-parametric geo-statistical models with location-specific random effects were fit to estimate the degree of zero inflation, over-dispersion, temporal and spatial correlation in the child HIV/TB mortality data. Spatial models showed more stable results with smaller standard errors in comparison to the non-spatial, the mean-based adjusted hazard ratios [AHR = exp(mean)] estimations were reported. The ages (interval start ages) were used in the analysis as the duration of follow-up (offset variables) until death, loss-to-follow up or survival between 1 and 5 years of age over the period 2000–2005. The outcome variable was treated as discrete count of child deaths due to HIV/TB (0 = Alive and 1 = Death). The outcome was treated as such to get more epidemiologically intuitive estimates for cohort data and to avoid identifiability problems associated with the logit link functions as highlighted earlier. Multivariable regression models were fit to account for spatial and temporal correlation; co-variables were added to the models to control for potential confounding in determining factors associated with child HIV/TB mortality (Pacheco et al., 2008). Poisson and Negative Binomial models were fit to the data as well as their zero inflated variants. The offset variable was fit using the piece wise exponential (p.e.m) with a first order autoregressive model temporal prior (see Fig. 4). Fig. 4 shows the log-baseline hazard function for selected models, showing a narrower credible interval for the spatial multivariable model in comparison to the non-spatial.

Four models were fit in the multivariable analyses these were the non-spatial zero inflated Poisson (ZIP) model, spatial zero inflated Poisson model, non-spatial zero inflated Negative Binomial (ZINB) model and spatial zero inflated Negative Binomial model. The first two were to cater for zero inflation and the last two were catering for zero inflation as well as over dispersion. The deviance information criteria (DIC) was used to measure model goodness of fit and the effective number of parameters $\left(p_{D}\right)$ was used to measure model complexity (Spiegelhalter et al., 2002). Generally the spatial models had higher DIC because of the spatial parameters and also the Negative Binomial models had greater DICs compared to the Poisson; this made it difficult to judge the best model based on the DIC alone. The $p_{D}$ which assesses model simplicity (parsimony) was then used to determine the most parsimonious model amongst these four. The zero inflated Negative Binomial spatial model had the least number of effective parameters and catered for over-dispersion, zero inflation and spatial random effects (see Table 1). We therefore focused on this model in the results and discussion sections.

The spatial multivariable analyses results showed several determinants of child HIV/TB mortality in the categories consistent with the non-spatial analyses. Several variables were statistically significant at the 5% level, these were either child, mother or household related. The results from the analyses are shown in tables 2 and 3 for the ZIP and ZINB, respectively. The ZIP models had five and six out of the sixteen variables significant in the non-spatial and spatial analysis, respectively, whereas the ZINB had eight and nine variables for non-spatial and spatial models, respectively. The final spatiotemporal ZINB model was considered the “best” model which caters well for zero inflation, over-dispersion and spatiotemporal confounding.

The final zero inflated spatial Negative Binomial model results showed these nine predictors of child mortality: gender, nationality of child, death of mother, gender of head of household, year, antenatal clinic visits, socio-economic status, birth order position and number of deaths in household. The boys were 36% more at risk of death compared to the girls adjusting for other variables and space-time confounders $\left(1.36=e^{0.311}\text{,}95\%\text{CI}[1.08\text{,}1.88]\right)$. There was a temporal effect on HIV/TB mortality risk for the year 2002 which was more than twice that of 2000 $\left(2.02=e^{0.719}\text{,}95\%\;\text{CI}[1.13\text{,}4.00]\right)$. South African children were almost twice better protected from HIV/TB death compared to the Mozambican children after adjusting for several variables $\left(0.51=e^{-0.683}\text{,}95\%\;\text{CI}[0.24\text{,}0.96]\right)$. Orphans with a deceased mother were almost thrice at risk of HIV/TB death compared to those whose mother’s were alive keeping other variables constant $\left(2.93=e^{1.075}\text{,}95\%\;\text{CI}[1.29\text{;}6.93]\right)$. Adjusting for other factors male headed households were 42% less likely to experience a child death in comparison to female headed households $\left(0.58=e^{-0.548}\text{,}95\%\;\text{CI}[0.45\text{;}0.75]\right)$.

For every increase by one visit to the antenatal clinic the adjusted risk of death reduced by 16% ($0.84=e^{-0.173}\text{,}95\%\;\text{CI}[0.81\text{;}0.89])$. The least economically poor households were 62% less likely to experience a child death compared to the most poor households ($0.38=e^{-0.966}\;\text{adjusted,}\;95\%\;\text{CI}[0.24\text{;}0.61])$. The birth order position was protective such that as this increased by one level the adjusted risk of child death decreased by 27% ($0.73=e^{-0.311}\text{,}95\%\;\text{CI}[0.58\text{;}0.89])$. Lastly as the number of household deaths increased by one the adjusted risk of child HIV/TB death increased two fold ($2.00=e^{-0.694}\text{,}95\%\;\text{CI}[1.12\text{;}3.33])$.

**Fig. 5:**
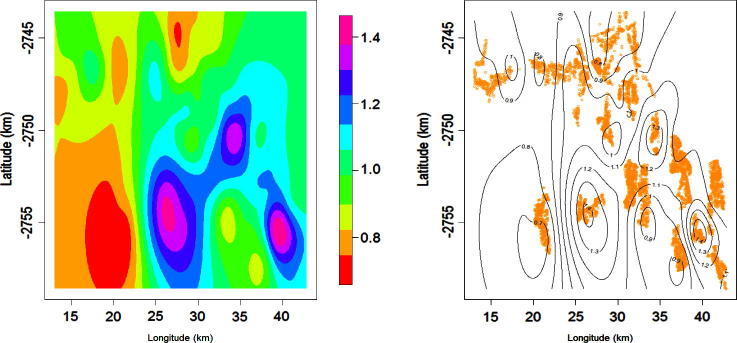
Posterior adjusted hazard rates (AHRs) smoothed map (left) and contour AHRs map (right) for child HIV/TB mortality 2000–2005.

Posterior risk estimates were displayed on smoothed maps showing the smoothed areas where child deaths occurred. Maps of posterior mean adjusted hazard rates (AHRs) show point estimates and extreme high and low risk areas (Ranta and Penttinen, 2000). Risk factors for child HIV/TB mortality adjusting for potential spatiotemporal confounders were: being a boy, a former foreigner, losing your mother, having a female head of house, being socio-economically disadvantaged, being born earlier and coming from households were many deaths occurred. Protective of child HIV/TB mortality were increasing number of clinic visit and coming from households with more people. Fig. 5 shows the maps of the households and the mean AHRs smoothed posterior maps adjusting for the aforementioned risk factors, spatial and temporal potential confounders.

## 4 Discussion and conclusions

In our study we found that maternal orphans experienced a threefold greater risk of death due to HIV/TB than children whose mothers were still living (AHR = 2.93, 95% CI[1.26; 6.93]). This was adjusted for potential spatiotemporal confounding, number of household deaths, birth order position and parity, child and mother’s nationality, gender of house head and gender of child, which were all significant risk factors. Increased antenatal clinic visits and cumulative total of adults in the house were protective of the child’s death. These risk factors are interrelated and fall into either child-specific, mother-related, household socio-economic status, temporal or spatial. Risk map analysis showed that there were three hotspots central, south easterly and south-westerly over the years 2000–2005; similar hot spots were seen for the year 2004 alone and also in another study considering only the areal (village) level and temporal random effects (Sartorius et al., 2011). The trends seen in this paper are consistent with other studies in South Africa, in the same site and elsewhere. A study in Agincourt showed that HIV/TB accounted for about a third of the under-five deaths from 2002 to 2005 (Tollman et al., 2008). A similar non-spatial study on the impact of parental presence on child mortality in Agincourt 1997–2005, showed that death of a mother was the most predictive of the child’s death (OR = 5.22, 95% CI[2.37; 11.54]) (Collinson et al., 2009). The study also showed that former Mozambican mothers’ children were more than a third at greater risk of mortality than the South Africans’ children (OR = 1.34, 95% CI[1.09; 1.65]). Similarly in rural Kwazulu-Natal child Province, mother’s death increased the mortality risk fourfold adjusting for child, household and mother’s variables (Argeseanu, 2004).

There are different schools of thoughts as to how the maternal orphan HIV/TB mortality trends can be explained, biological and socio-economical. Mothers who die due to AIDS, may have transmitted the virus to their children, regardless of the stage at which this occured (in utero, intrapartum or postnatal). Support programs currently favour local South African mothers, making it more difficult for breast feeding foreign mother’s to access available services and resources. Socially, if the mother (who will most likely be the remaining bread winner) dies, this leaves AIDS orphans with no caregiver or with grandparents who can no longer work. This contributes to the deaths of children due to poor nutrition, weakened immune system and lack of adequate support. Although we concentrated on HIV as the main cause of death for these under five children, TB may have played a significant role leading to death. However the verbal autopsy technique used to determine cause of death does not distinguish these very well for children. Although Agincourt is in rural South Africa, the use of available health facilities seems to be increasing; using antenatal clinic visits as a proxy.

The other risk factors were mainly related to poverty as direct proxies or indirect consequences of it. We found that poorer socio-economic households had higher child HIV/TB mortality rates, a finding which corresponds with a study in rural Kwazulu-Natal Province (Ndirangu et al., 2010). Poverty is a major contributor to child deaths in Africa, every 3.6 s one person dies of starvation, usually a child under the age of 5. One of the Millennium Development Goals (MDGs) is to: “Eradicate extreme poverty and hunger” (Eftim et al., 2008; Sachs, 2010). The child support grant provided by the South African government is an amount of ZAR260 p/m (US$38), is often all that some families in the study area have to live on. The elderly get an old-age pension of ZAR1140 p/m (US$163) which can be the main source of livelihood of families. A study showed that female old-age pensioners cared for children and improved the girl child’s likelihood to go to school in Agincourt (Case and Menendez, 2007). These studies reveal gender differentials in access to services, and schooling.

Controlling for potential confounding in epidemiological analyses gives strength to the estimates derived from such modelling procedures. Our modelling approach caters for spatial and temporal confounders and also for large zero inflated spatiotemporal sparse data. Spatiotemporal Bayesian modelling provided good estimates and maps which can guide interventional programmes to be more effective in addressing HIV/TB. There are some modelling limitations which if addressed in future research will improve on the epidemiological modelling. The temporal component was discretized into whole years; this could have been modelled as continuous and allow for formulation of time varying covariates and spatiotemporal interactions. As a way of dealing with identifiability issues and getting more epidemiologically correct estimates; members of the exponential (log link) family were used, other less flexible family of distributions could also be used for sensitivity testing. Mostly non-informative priors have been used; experts opinion could be sought to build and apply more informative priors in our modelling. In dealing with the “big N” problem several approaches can be used. The package BayesX, whilst it cannot address all the modelling issues, provides very useful Bayesian spatiotemporal modelling algorithms which are applicable even in a resource scare environment.

Our results provide evidence useful for policy makers advocating for better access to health-care and child support grants especially for non-South Africans, increase and diversification of HIV mother to child transmission awareness programmes in rural areas. This study provides evidence to support the South African government’s revised HIV policy effected on 1 April 2010. The policy allows HIV positive mothers to be on ante-retro-virals with a CD4 count of 350 (national cut off is 200) or less, symptomatic HIV regardless of CD4 and also pregnant mothers from 14 gestational weeks onwards. Infants can now be tested without the mother’s consent to save the child (Zuma, 2009). Child survival is dependent on the survival of the mother, hence the need to strengthen anti-retro-viral treatment. South African policy supporting maternal survival need to be strengthened. In an address to the nation the South African president said: “One of the most important results of the roll-out of anti-retro-viral therapy among the general population will be the extension of the lives of AIDS sick parents leading to a dramatic decline in the number of orphans” (Bradshaw et al., 2002). The main challenge as with many other policies is implementation. We suggest targeting endeavours to reduce mother-to-child transmissions by effectively implementing interventions that reach rural villages and the poorest households which are often neglected.

## Authors’ contributions

E.M. drafted the manuscript, did the statistical analysis and wrote this paper. P.V. and K.K. gave their expert advice on methodology and epidemiology aspects of this paper, respectively.

## Competing interests

The authors declare that they have no competing interests.

## Figures and Tables

**Table 1:** Model goodness of fit non-spatial/spatial zero inflated Poisson and Negative Binomial models.

	Non-spatial zero inflated Poisson	nsZIP	Spatial zero inflated Poisson	sZIP	Non-spatial zero inflated Negative Binomial	nsZINB	Spatial zero inflated Negative Binomial	sZINB
	Un-standardized	Saturated	Un-standardized	Saturated	Un-standardized	Saturated	Un-standardized	Saturated
$\overset{¯}{D}$	649.16	273.16	664.3	288.33	1674.77	1252.4	1865.41	1228.41
*pD*	246.51	246.51	247.7	247.7	83.86	104.72	47.95	36.23
*DIC*	1142.2	766.19	1159.79	783.79	1842.49	1461.86	1961.32	1300.88

**Table 2:** Non-spatial and spatial multivariable zero inflated Poisson models.

Variable	Category	Descriptive, *n* (%) *N* = 16,844	Non-spatial zero inflated Poisson					nsZIP	Spatial zero inflated Poisson					sZIP
Mean	SD	2.5%	Median	97.50%	AHR	Mean	SD	2.5%	Median	97.50%	AHR
Gender	Female (ref)	8506 (50.50)												
	Male	8838 (49.50)	0.415	0.25	−0.12	0.42	0.93	1.51	0.38	0.25	−0.11	0.39	0.88	1.46
Year	2000 (ref)	3360 (19.95)												
	2001	2826 (16.78)	0.49	0.30	−0.14	0.50	1.10	1.63	0.50	0.30	−0.10	0.52	1.02	1.65
	2002	2633 (15.63)	0.837⁎	0.37	0.16	0.86	0.54	2.31	0.81⁎	0.333	0.17	0.81	1.46	2.25
	2003	2496 (14.82)	0.56	0.43	−0.21	0.53	1.43	1.75	0.56	0.40	−0.32	0.58	1.27	1.75
	2004	3002 (17.82)	0.15	0.34	−0.50	0.15	0.80	1.16	0.17	0.32	−0.52	0.17	0.80	1.19
	2005	2527 (15.00)	−0.22	0.47	−1.14	−0.20	0.61	0.80	−0.21	0.45	−1.07	−0.20	0.65	0.81
Child’s nationality	Mozambican (ref)	6242 (37.06)												
	South African	10,602 (62.94)	−0.82	0.65	−2.11	−0.86	0.34	0.44	−0.70	0.53	−1.65	−0.69	0.36	0.50
Mother’s nationality	South African (ref)	10,883 (64.61)												
	Mozambican	5961 (35.39)	−0.11	0.67	−1.56	−0.11	1.25	0.90	−0.05	0.54	−1.07	−0.05	1.00	0.95
Mother’s age at birth	Mean years ± SD	29.00 ± 7.60	0.01	0.02	−0.02	0.01	0.04	1.01	0.01	0.02	−0.03	0.01	0.04	1.01
Mother’s status	Alive (ref)	16,736 (99.36)												
	Deceased	108 (0.64)	2.665⁎	0.81	0.90	2.74	3.90	14.37	2.456⁎	0.63	1.15	2.46	3.74	11.66
Father’s status	Alive (ref)	16,531 (98.14)												
	Deceased	313 (1.86)	0.98	0.66	−0.33	1.00	2.33	2.66	1.081⁎	0.49	0.06	1.09	2.04	2.95
Household head gender	Female (ref)	3012 (33.98)												
	Male	5851 (66.02)	−0.49	0.26	−1.04	−0.50	0.01	0.61	−0.45	0.25	−0.96	0.44	0.02	0.64
Antenatal visits	Mean visits ± SD	4.04 ± 2.42	−0.185⁎	0.05	−0.29	−0.19	−0.09	0.83	−0.184⁎	0.05	−0.29	−0.18	−0.90	0.83
Pregnancy parity	Mean ± SD	2.104 ± 1.396	0.25	0.22	−0.14	0.24	0.67	1.28	0.25	0.21	−0.16	0.25	0.67	1.28
Household socio-economic status	Most poor (ref)	1566 (17.67)												
	Very poor	1701 (19.19)	−0.17	0.32	−0.83	−0.17	0.41	0.84	−0.22	0.30	−0.82	−0.22	0.36	0.80
	Poor	1833 (20.68)	−0.20	0.35	−0.84	−0.20	0.51	0.82	−0.28	0.29	−0.85	−0.29	0.27	0.76
	Moderately poor	1905 (21.49)	−0.26	0.33	−1.03	−0.27	0.36	0.77	−0.30	0.32	−0.95	−0.29	0.29	0.74
	Least poor	1858 (20.97)	−0.856⁎	0.42	−1.60	−0.84	−0.04	0.42	−0.941⁎	0.42	−1.83	−0.94	−0.13	0.39
Cumulative household deaths	Mean ± SD	0.252 ± 0.553	−0.16	0.25	−0.68	−0.17	0.29	0.85	−0.10	0.24	−0.62	−0.13	0.30	0.90
Parity at birth	Mean ± SD	1.569 ± 0.856	−0.369⁎	0.20	−0.77	−0.35	−0.03	0.69	−0.37⁎	0.18	−0.74	−0.37	−0.02	0.69
Total living household	Mean ± SD	1.569 ± 0.884	0.01	0.02	−0.04	0.01	0.06	1.01	0.00	0.02	−0.04	0.00	0.04	1.00
Number of deaths in household	Mean ± SD	0.047 ± 0.222	0.854	0.50	−0.11	0.86	1.80	2.35	0.89	0.48	−0.17	0.92	1.79	2.44
Total live births	Mean ± SD	2.030 ± 1.855	−0.21	0.18	−0.60	−0.21	0.13	0.81	−0.22	0.18	−0.61	−0.21	0.12	0.80

⁎ Significant at 5% level of significance.

**Table 3:** Non-spatial and spatial multivariable zero inflated Negative Binomial models.

Variable	Category *N* = 16,844	Descriptive, *n* (%)	Non-spatial zero inflated Negative Binomial					nsZINB	Spatial zero inflated Negative Binomial					sZINB
Mean	SD	2.5%	Median	97.50%	AHR	Mean	SD	2.5%	Median	97.50%	AHR
Gender	Female (ref)	8506 (50.50)												
	Male	8338 (49.50)	0.31⁎	0.14	0.07	0.28	0.62	1.32	0.326⁎	0.17	0.08	0.31	0.63	1.36
Year	2000 (ref)	3360 (19.95)												
	2001	2826 (16.78)	0.46	0.27	−0.05	0.48	1.05	1.62	0.55	0.23	−0.06	0.58	0.92	1.78
	2002	2633 (15.63)	0.74⁎	0.24	0.16	0.79	1.14	2.20	0.762⁎	0.31	0.12	0.72	1.38	2.05
	2003	2496 (14.82)	0.39	0.32	−0.19	0.40	1.01	1.48	0.45	0.31	−0.14	0.45	1.02	1.56
	2004	3002 (17.82)	0.00	0.25	−0.42	0.00	0.46	1.00	0.03	0.24	−0.40	0.03	0.49	1.03
	2005	2527 (15.00)	−0.54	0.41	−1.27	−0.53	0.26	0.59	−0.43	0.32	−1.08	−0.41	0.12	0.67
Child’s nationality	Mozambican (ref)	6242 (37.06)												
	South African	10,602 (62.94)	−0.41	0.32	−1.02	−0.35	0.09	0.71	−0.643⁎	0.31	−1.43	−0.68	−0.04	0.51
Mother’s nationality	South African (ref)	10,883 (64.61)												
	Mozambican	5961 (35.39)	0.05	0.32	−0.59	0.06	0.57	1.06	−0.12	0.33	−0.68	−0.19	0.57	0.83
Mother’s age at birth	Mean years ± SD	2900 ± 7.60	0.01	0.01	−0.01	0.01	0.05	1.01	0.01	0.01	−0.01	0.01	0.03	1.01
Mother’s status	Alive (ref)	16,531 (99.36)												
	Deceased	108 (0.64)	1.479⁎	0.36	0.63	1.52	2.15	4.55	1.09⁎	0.47	0.25	1.08	1.94	2.93
Father’s status	Alive (ref)	16,531 (98.14)												
	Deceased	313 (1.86)	0.17	0.32	−0.55	0.24	0.70	1.27	0.11	0.44	−0.82	0.16	0.82	1.17
Household head gender	Female (ref)	3017 (33.98)												
	Male	5851 (66.02)	−0.449⁎	0.16	−0.70	−0.49	−0.15	0.62	−0.54⁎	0.14	−0.79	−0.55	−0.29	0.58
Antenatal Visits	Mean visits ± SD	4.04 ± 2.42	−0.156⁎	0.03	−0.21	−0.16	−0.09	0.85	−0.17⁎	0.03	−0.22	−0.17	−0.12	0.84
Pregnancy parity	Mean ± SD	2.104 ± 1.396	−0.04	0.11	−0.24	−0.03	0.18	0.97	0.08	0.11	−0.14	0.08	0.26	1.09
Household socio-economic status	Most poor (ref)	1566 (17.67)												
	Very poor	1701 (19.19)	−0.32	0.18	−0.65	−0.32	0.08	0.72	−0.36	0.24	−0.81	−0.31	0.09	0.73
	Poor	1833 (20.68)	−0.37	0.20	−0.77	−0.38	0.05	0.68	−0.41	0.21	−0.79	−0.43	0.02	0.65
	Moderately poor	1905 (21.49)	−0.23	0.24	−0.78	−0.22	0.18	0.80	−0.28	0.24	−0.75	−0.26	0.22	0.77
	Least poor	1858 (20.97)	−0.945⁎	0.33	−1.57	−0.97	−0.28	0.38	−0.99⁎	0.27	−1.44	−0.97	−0.49	0.38
Cumulative household deaths	Mean ± SD	0.252 ± 0.553	−0.10	0.14	−0.38	−0.09	0.16	0.92	−0.14	0.16	−0.48	−0.15	0.14	0.86
Parity at birth	Mean ± SD	1.569 ± 0.856	−0.291⁎	0.12	−0.54	−0.31	−0.04	0.74	−0.314⁎	0.11	−0.55	−0.31	−0.12	0.73
Total living household	Mean ± SD	1.569 ± 0.884	0.01	0.01	−0.01	0.01	0.04	1.01	0.01	0.01	−0.01	0.01	0.04	1.01
Number of deaths in household	Mean ± SD	0.047 ± 0.222	0.703⁎	0.27	0.14	0.71	1.16	2.03	0.681⁎	0.29	0.12	0.69	1.20	2.00
Total live births	Mean ± SD	2.030 ± 1.855	0.03	0.09	−0.11	0.02	0.23	1.02	−0.06	0.09	−0.26	−0.07	0.08	0.94

⁎ Significant at 5% level of significance.

## Appendix A Zero inflated Poisson and Negative Binomial Hierarchical Bayesian models

Observational outcome data can be modelled by different known statistical distributions including the flexible exponential family. Mortality outcomes of the count of deaths can be modelled using the basic log-linear Poisson model were the outcomes $y_{i}|X_{i}∼\mathrm{Po}(\lambda_{i})$ are conditionally independent with mean and variance $\lambda_{i}=\exp(\eta_{i})$ with linear predictor $\eta_{i}=X_{i}\beta$. The commonly applied logistic regression models which have logit link functions have been seen to cause identifiability problems, resulting in unstable model especially for spatial models (Agarwal et al., 2002). We therefore treated the outcome death as a discrete (count) variable since this provides better estimates of prevalence and relative risk compared to the odds ratio which results from treating it as binary outcome (Barros and Hirakata, 2003; Fekedulegn et al., 2010). The Poisson model can also incorporate the exposure time by introducing piece-wise exponential offsets o_i_ resulting in $y_{i}|X_{i}∼\mathrm{Po}(\mu_{i})\text{,}\mu_{i}=\exp(o_{i}+\eta_{i})$ and in log-linear form $y_{i}|\eta_{i}∼\mathrm{Po}(\mu_{i})$ (Brezger and Lang, 2006). In this paper, we extend this model in three dimensions to address different inherent problems in our hierarchical data structure. Firstly the problem of over-dispersion when the mean is less than the variance which can be addressed by use of conditionally independent Negative Binomial (NB) distribution (Ridout et al., 2001). We then extend our model incorporate the dispersion parameter $\delta >0\text{,}y_{i}|\eta_{i}\text{,}\delta ∼\mathrm{NB}(\mu_{i}\text{,}\delta )$. Secondly we have the problem of excess of zeros known as zero inflation, this is addressed by use of a latent process that inflates the number of zero points with a weighting θ for extra zeros and 1-θ for the ZIP and ZINB where 0<θ<1. We then obtain the conditionally independent zero inflated Poisson $y_{i}|\eta_{i}\text{,}\theta ∼\mathrm{ZIP}(\mu_{i}\text{,}\theta )$ and zero inflated Negative Binomial (ZINB) $y_{i}|\eta_{i}\text{,}\delta \text{,}\theta ∼\mathrm{ZINB}(\mu_{i}\text{,}\delta \text{,}\theta )$ whose hierarchical model structures and likelihoods are shown below. Thirdly modelling which is flexible to include; time varying covariates, temporal random effects, spatial random effects, non-linear covariates and desired interactions. This was done by extending the linear predictor $\eta_{i}=X_{i}\beta$ to a structured additive predictor:

$$\eta_{i}=\overset{p}{\underset{j=1}{\sum }}f_{j}(z_{\mathrm{ij}})+X_{i}\beta +f_{{\mathrm{spat}}_{i}}(s_{k})+\varepsilon_{s_{k}}$$

where $f_{i}(z_{\mathrm{ij}})$ are nonlinear effects of continuous covariate $z_{\mathrm{ij}}\text{,}X_{i}$ is the linear (fixed) effects design matrix and vector of parameters $\beta \text{,}f_{{\mathrm{spat}}_{i}}(s_{k})$ is the structured random spatial at household $s_{k}\in$s (all households with children in the area) and $\varepsilon_{s_{k}}$ being the unstructured non-spatial random effect. Due to the complexity of the spatiotemporal data (correlated in time and space) and their large size. Statistical methods applied to spatial data which have spatial autocorrelation often underestimate the standard error and thus the statistical significance is overestimated (Cressie, 1993). We used the Bayesian estimation called the Markov Chain Monte Carlo (MCMC), which require Gibbs sampling and Metropolis Hastings within Gibbs (Banerjee et al., 2004). Modelling spatial longitudinal data which are highly auto-correlated has been made possible by introduction of the Hierarchical Bayesian (HB) modelling approach. This decomposes a complex problem into a series of simpler conditional levels such that at any given level in the hierarchy inference conditionally relies on hypotheses made at higher levels (Banerjee et al., 2004). The HB model constitutes of four levels: firstly the data level which specifies the conditional distribution of data given parameters i.e $y_{i}$; secondly a process level which specifies the conditional distributions of process given their parameters (these are usually logit and log functions of Generalised Linear Models); thirdly a parameter level with conditional parameter distributions given priors and lastly hyper-parameter level which specifies the prior distributions for hyper-parameters. The posterior distribution can then be used for full Bayesian inference of the ZIP and ZINB models given the following full conditional distributions. To estimate the parameters Gibbs sampling is used by sampling from the full conditional distributions for posteriors with known closed forms (Casella and George, 1992). For those without the closed form we use the Metropolis Hastings (MH) random walk algorithms (Browne and Draper, 2000).

### A.1 Zero inflated Poisson model

#### A.1.1 Level 1: Data

The ZIP with a semi-parametric predictor offers a flexible way to model the zero inflation parameter (θ).

$y_{i}|\eta_{i}\theta ∼\text{ZIP}(\mu_{i}\theta )$ with probability density function

$$P(y_{i}|\eta_{i}\text{,}\theta )=\left\{\begin{array}{cc} \theta +(1-\theta )\frac{\exp(-\mu_{i})\mu_{i}^{y_{i}}}{y_{i}^{!}} & \mathrm{if}\;y_{i}=0\\ (1-\theta )\frac{\exp(-\mu_{i})\mu_{i}^{y_{i}}}{y_{i}^{!}} & \mathrm{if}\;y_{i}\;\neq \;0 \end{array}\right)$$

With expectation $E(y_{i}|\eta_{i}\text{,}\theta )=(1-\theta )\mu_{i}$ and variance $V(y_{i}\eta_{i}\text{,}\theta )=(1-\theta )(\mu_{i})+\theta (1-\theta )\mu_{i}^{2}$.

#### A.1.2 Level 2: Processes

$$\begin{array}{cc} & \eta_{i}=X_{i}^{\prime }\beta +f_{{\mathrm{spat}}_{i}}(s_{k})+\varepsilon_{s_{k}}\text{;}\\ & \theta ∼\mathrm{Uniform}[0\text{,}1] \end{array}$$

$X_{i}$ is the linear (fixed) effects design matrix and vector of parameters $\beta \text{,}f_{{\mathrm{spat}}_{i}}(s_{k})$ is the structured random spatial effect and $\varepsilon_{s_{k}}$ being the unstructured non-spatial random effect.

#### A.1.3 Level 3: Parameters

Priors for $P(\beta )\text{,}P(f_{{\mathrm{spat}}_{i}}|\tau_{\mathrm{spat}}^{2})\text{,}P(\varepsilon_{s_{k}}|\tau_{\mathrm{error}}^{2})\text{,}P(\theta )$. Uniform priors for P(β) and P(θ). Multivariate Gaussian random filed for $P(f_{{\mathrm{spat}}_{i}}|\tau_{\mathrm{spat}}^{2})$ and $P(\varepsilon_{s_{k}}|\tau_{\mathrm{error}}^{2})$ are Gaussian independent and identically distributed (i.i.d) random effects.

#### A.1.4 Level 4: Hyper-parameters and likelihood

Hyperpriors for $P(\tau_{\mathrm{spat}}^{2})\text{,}P(\tau_{\mathrm{error}}^{2})$ and $g(\tau^{2})$ are all Inverse Gamma $∼\mathrm{IG}(a^{*}\text{,}b^{*})$; with hyper parameters *a*^∗^ = *b*^∗^ = 0.001 (Brezger and Lang, 2006). After some computations the likelihood of the ZIP models is:

$$L(y_{i}|\eta_{i}\text{,}\theta )=\overset{n}{\underset{i=1}{\prod }}P(y_{i}|\eta_{i}\text{,}\theta )\propto \exp\left\{\underset{y_{i}=0}{\sum }\log(\theta +(1-\theta )\exp(-\mu_{i}))+W_{o}\;\log\;(1-\theta )+\underset{y_{i}\;\neq \;0}{\sum }(-\mu_{i}+y_{i}\log(\mu_{i}))\right\}$$

where $W_{0}$ is the number of units with strictly positive responses.

### A.2 Zero inflated Negative Binomial model

#### A.2.1 Level 1: Data

The ZINB with a semi-parametric predictor offers a flexible way to model the variability of the data catering for over-dispersion (δ), zero inflation (θ).

$y_{i}|\eta_{i}\text{,}\delta \text{,}\theta ∼\mathrm{ZINB}(\mu_{i}\text{,}\delta \text{,}\theta )$ with probability density function

$$P(y_{i}|\eta_{i}\text{,}\delta \text{,}\eta )=\left\{\begin{array}{cc} \theta +(1-\theta ){\left(\frac{\delta }{\delta +\mu_{i}}\right)}^{\delta } & \mathrm{if}\;y_{i}=0\\ (1-\theta )\frac{\Gamma (y_{i}+\delta )}{\Gamma (y_{i}+1)\Gamma (\delta )}{\left(\frac{\delta }{\delta +\mu_{i}}\right)}^{\delta }{\left(\frac{\mu_{i}}{\delta +\mu_{i}}\right)}^{y_{i}} & \mathrm{if}\;y_{i}\;\neq \;0 \end{array}\right)$$

With expectation $E(y_{i}|\eta_{i}\text{,}\theta )=(1-\theta )\mu_{i}$ and variance $V(y_{i}|\eta_{i}\text{,}\delta \text{,}\theta )=(1-\theta )(\mu_{i}+\frac{\mu_{i}^{2}}{\delta })+\theta (1-\theta )\mu_{i}^{2}$.

#### A.2.2 Level 2: Processes

$$\begin{array}{cc} & \eta_{i}=X_{i}^{\prime }\beta +f_{{\mathrm{spat}}_{i}}(s_{k})+\varepsilon_{s_{k}}\text{;}\\ & \delta |b∼\mathrm{Gamma}(a\text{,}b)\text{;}\theta ∼\mathrm{Uniform}[0\text{,}1] \end{array}$$

$X_{i}$ is the linear (fixed) effects design matrix and vector of parameters β, $f_{{\mathrm{spat}}_{i}}(s_{k})$ is the structured random spatial effect and $\varepsilon_{s_{k}}$ being the unstructured non-spatial random effect. We set *a* = 1 and assume a Gamma hyper-prior for b∼Gamma(α1,α2).

#### A.2.3 Level 3: Parameters

Priors for $P(\beta )\text{,}P(f_{{\mathrm{spat}}_{i}}|\tau_{\mathrm{spat}}^{2})\text{,}P(\varepsilon_{s_{k}}|\tau_{\mathrm{error}}^{2}\text{,}P(\theta )\text{,}P(\delta |b))$. Uniform priors for P(β) and P(θ), Gamma priors for P(δ|b). Multivariate Gaussian random field (GRF) priors for $P(f_{{\mathrm{spat}}_{i}}|\tau_{\mathrm{spat}}^{2})$ and $P(\varepsilon_{s_{k}}|\tau_{\mathrm{error}}^{2})$ are Gaussian independent and identically distributed (i.i.d) random effects.

#### A.2.4 Level 4: Hyper-parameters and likelihood

Hyperpriors for $P(\tau_{\mathrm{spat}}^{2})$, $P(\tau_{\mathrm{error}}^{2})$ and P(b) are all Inverse Gamma $∼\mathrm{IG}(a^{*}\text{,}b^{*})$; with hyper parameters *a*^∗^ = *b*^∗^ = 0.001 (Brezger and Lang, 2006). After some computations the likelihood of the ZINB models is:

$$L(y_{i}|\eta_{i}\text{,}\delta \text{,}\theta )=\overset{n}{\underset{i=1}{\prod }}P(y_{i}|\eta_{i}\text{,}\delta \text{,}\theta )\infty \;\propto \exp\;\left\{\underset{y_{i}=0}{\sum }\;\log\;\left(\theta +(1-\theta ){\left(\frac{\delta }{\delta +\mu_{i}}\right)}^{\delta }\right)\right)+W_{o}(\log(1-\theta )-\log(\Gamma (\delta ))+\delta \log(\delta ))+\underset{y_{i}\;\neq \;0}{\sum }\log(\Gamma (y_{i}+\delta ))+y_{i}\log(\mu_{i})-(y_{i}\left(+\delta )\log(\delta +\mu_{i})\right\}$$

where $W_{0}$ is the number of units with strictly positive responses.

## Footnotes

1 A16 = respiratory tuberculosis; not confirmed bacteriologically or histologically; A17 = tuberculosis of nervous system; A18 = tuberculosis of other organs; A19 = miliary tuberculosis.

2 B20 = human immunodeficiency virus (HIV) disease resulting in infectious and parasitic diseases; B21 = human immunodeficiency virus (HIV) disease resulting in malignant neoplasms; B22 = human immunodeficiency virus (HIV) disease resulting in other specified diseases; B23 = human immunodeficiency virus (HIV) disease resulting in other conditions; B24 = unspecified human immunodeficiency virus (HIV) disease.

3 For interpretation of color in Fig. 3, the reader is referred to the web version of this article, these appear light grey in the black and white printed version.
